# Synergistic activity of tafasitamab and metronomic chemotherapy on diffuse large B-cell lymphoma through inhibition of the AKT/mTOR signaling pathway

**DOI:** 10.1038/s41598-025-95476-y

**Published:** 2025-04-03

**Authors:** Marta Banchi, Maria Christina Cox, Paola Orlandi, Arianna Bandini, Fabio Stefanelli, Silvio Chericoni, Guido Bocci

**Affiliations:** 1 Department of Translational Research and of New Surgical and Medical Technologies, University of Pisa, Via Roma 55, 56126 Pisa, Italy; 2 Haematology Unit, Fondazione Policlinico Tor Vergata, Rome, Italy; 3 Department of Clinical and Experimental Medicine, University of Pisa, Pisa, Italy; 4 Department of Surgical, Medical and Molecular Pathology and Critical Care Medicine, University of Pisa, Pisa, Italy

**Keywords:** Diffuse large B-cell lymphoma, Metronomic chemotherapy, Vinorelbine, Etoposide, Tafasitamab, B-cell lymphoma, Immunotherapy, Cell growth, Apoptosis, Cell signalling

## Abstract

Tafasitamab is a novel humanized anti-CD19 monoclonal antibody, designed for the treatment of B-cell malignancies. Our study aims to enhance the direct, non-immune-mediated, activity of tafasitamab (TAFA) with the combination of metronomic chemotherapy (mCHEMO), including vinorelbine (mVNR) and etoposide (mETO), in preclinical models of diffuse large B-cell lymphoma (DLBCL). In vitro*,* the 144 h exposure of thrice-weekly mVNR, daily mETO, and single-dose TAFA significantly inhibited the viability of human CD19^+^ DLBCL cell lines (i.e., Toledo, OCI-LY3, and SU-DHL10) in a concentration-dependent manner. In all cell lines, the concomitant treatment with TAFA and mVNR or mETO showed a marked synergism, except for TAFA + mETO on SU-DHL10 cells. The TAFA + mCHEMO treatments promoted apoptosis, and the TAFA + mVNR combination significantly inhibited, already after 24 h, the phosphorylation of GSK3α/β, mTOR, p70S6K, RPS6, and TSC2 proteins in DLBCL cells. TAFA significantly increased the VNR and ETO intracellular concentrations in all DLBCL cells after 24 h, except for ETO levels in SU-DHL10. The TAFA + mCHEMO treatment strongly reduced the *ABCB1*, *ABCG2,* and *c-MYC* gene expression in SU-DHL10 cells. In vivo, the TAFA + mVNR combination was well tolerated, significantly reduced the volumes of subcutaneous DLBCL masses, and increased the overall survival of mice affected by systemic DLBCL. We report additional mechanisms to enhance the direct activity of TAFA with mCHEMO synergistically in DLBCL cells in vitro and in vivo, suggesting the use of this combination schedule into future clinical trials.

## Introduction

Diffuse large B-cell lymphoma (DLBCL) is the most common type of aggressive non-Hodgkin lymphoma (NHL) worldwide^1^. Although standard chemotherapy remains the pillar of NHL treatment, in approximately one-third of DLBCL patients, the response to conventional treatment is not durable, relapse is frequent, and the prognosis is generally poor^2^.

Tafasitamab, a novel humanized, anti-CD19 monoclonal antibody (mAb) with an engineered constant fragment (Fc)-domain (i.e., two amino acid substitutions within the Fc region), has recently been designed to enhance FcγRIIIa binding affinity, for the treatment of B-cell malignancies^3^. It exhibits potent tumor cytotoxicity with both a direct mechanism and an indirect one, through effector function cells, such as antibody-dependent cell-mediated cytotoxicity (ADCC) and antibody-dependent cellular phagocytosis (ADCP)^4^. An open-label, noncomparative, multinational, phase II study of intravenous tafasitamab (12 mg/kg) in combination with oral lenalidomide (25 mg/kg) evidenced promising clinical activity and a durable response in adults with relapsed or refractory DLBCL after one to three systemic regimens (including at least one anti-CD20 therapy) and who were not candidates for high-dose chemotherapy and subsequent autologous stem cell transplant (ASCT)^5^.

CD19 plays a crucial role in the survival and proliferation of normal B cells. The signaling pathway initiated by the engagement of the CD19 cell surface receptor and its interaction with the B-cell receptor (BCR) complex is clearly described^6^. Key protein kinases associated with the CD19 complex include members of the Src family (Lyn, Fyn), Ras family, Abl, Btk, adapter molecules (Vav, Grb2), and PI3K^7^. Additionally, CD19 is essential for optimal MHC class II-mediated signaling, by modulating tyrosine phosphorylation and Akt signaling pathway^8^.

Metronomic chemotherapy (mCHEMO) can be defined as the frequent, regular administration of doses that maintain low, but active plasma concentrations of chemotherapeutic drugs with a good tolerability^9^. The major advantages of mCHEMO could be summarized with the following characteristics: 1) multiple mechanisms of action^10^, 2) easiness to combine with target therapies^11^, 3) low toxicity, 4) oral administration, and, therefore, 5) the feasibility of a more comfortable and inexpensive home-based treatment^12^. Furthermore, mCHEMO holds the potential to widen the chance of NHL cure and of improving patients’ care^13^, especially in the elderly/vulnerable population. Recently, the metronomic all-oral DEVEC schedule, based on prednisone [Deltacortene®, DE], vinorelbine [V], etoposide [E] and cyclophosphamide [C], + /- rituximab [R] was experimented in treatment naïve and relapsed/refractory elderly subjects, with B and T-cell aggressive NHL^14–16^. The response, progression free and overall survival rates compared with a real life setting of patients treated with standard intravenous chemotherapy were very promising. As a matter of fact, the combination of mCHEMO with novel drugs is still largely unexplored in NHL^11^. Conversely, most “maximum-tolerated dose-chemotherapy plus targeted therapies” combinations have not met expectations because of lack of synergy or excessive toxicity of the combination^17–19^.

Our aims in this study were to: 1) to evaluate and enhance the direct, non-immune-mediated, activity of tafasitamab in concomitant combination with metronomic vinorelbine (mVNR) and etoposide (mETO) on human DLBCL cells and 2) to translate in mouse xenograft models the antitumor efficacy of the combination, testing the mice overall survival.

## Materials and methods

### Materials, drugs and cell lines

RPMI-1640 medium (catalogue #R8758), fetal bovine serum (FBS, catalogue #F7524), antibiotics (catalogue #P4333) and all the other cited reagents were purchased from Sigma Aldrich SRL (Milan, Italy). Sterile plastics for cell culture were supplied by Costar (Cambridge, MA, USA). The human CD19^+^ DLBCL cell line Toledo (CRL-2631) and CD19^−^ cutaneous T-cell lymphoma (CTCL) cell line HH (CRL-2105) were obtained from the American Type Culture Collection (ATCC, Manassas, VA, USA). The human CD19 + DLBCL cell lines OCI-LY3 (ACC 761) and SU-DHL10 (ACC 576) were purchased from DMSZ (Leibniz Institute DSMZ-German Collection of Microorganisms and Cell Cultures GmbH, Braunschweig, Germany). DLBCL cells were maintained in an 80% RPMI-1640 medium supplemented with 20% heat-inactivated FBS, and antibiotics. The cells were routinely maintained in tissue culture flasks and kept in a humidified atmosphere of 5% CO_2_ at 37 °C. Cells were used for experiments at the fourth passage.

Vinorelbine (VNR, catalogue #S4269) and etoposide (ETO, catalogue #S1225) were obtained from Selleckchem (DBA Italia, Milan, Italy) and were diluted from a 10 mM stock solution (in 100% dimethylsulfoxide) for in vitro studies. Tafasitamab was kindly provided by Incyte (Morges, Switzerland) and diluted in sterile water from a 180 μM stock solution (in water for injection) for in vitro studies. Vehicle-treated controls received the same concentration of a human non-targeting IgG1 isotype control (catalogue #BE0297, BioXCell, DBA Italia, Milan, Italy) as cells of the highest concentration of tafasitamab, and the same concentration of dimethylsulfoxide (catalogue #D2650) in the media as did cells that were treated with the highest concentration of ETO.

### In vitro experiments

#### Cell cytotoxicity assay

HH, Toledo, OCI-LY3 and SU-DHL10 cells (2 × 10^4^) were seeded in 24-well plates and allowed to grow in suspension overnight. DLBCL cell lines were first exposed for 144 h to a human IgG1 isotype control (1–1,800 nM) to test the absence of cytotoxic activity and its use as negative control for all following experiments. CD19^−^ HH cells were then treated with tafasitamab (1–1,800 nM) or with vehicle alone to test tafasitamab specificity against CD19. CD19^+^ DLBCL cells were then treated for 144 h with mVNR (0.001–10 nM), mETO (0.025–100 nM), and tafasitamab (1–1,800 nM) or with vehicle alone (Supplementary Fig. S1). VNR or ETO were added every 48 h or 24 h, respectively, to mimic the clinical schedules. A single dose of tafasitamab was added at day one of treatment (Supplementary Fig. S1).

At the end of the treatment, the viable cells, assessed by trypan blue (catalogue #T8154) dye exclusion, were counted with a hemocytometer. The drug concentrations that reduced cell count by 50% (IC_50_) *versus* control cells were calculated by nonlinear regression fit of the mean values of data, obtained in triplicate experiments with at least 9 wells for each concentration, using GraphPad Prism software package version 9.0 (GraphPad Software, USA).

#### In vitro assessment of synergism between tafasitamab and metronomic vinorelbine or etoposide on DLBCL cells

The simultaneous exposure of mVNR (0.001–10 nM) or mETO (0.025–100 nM) combined with tafasitamab (1–1,800 nM) was explored on all the DLBCL cell lines at a fixed molar concentration ratio of 1:1000 or 1:100, respectively, for 144 h. To evaluate the level of interaction (synergistic, additive, or antagonistic) between mVNR and/or mETO and tafasitamab, the method proposed by Chou^20^ and the Loewe additivity model^14^ were followed. Briefly, synergism was calculated based on the multiple drug-effect equation and quantified by the combination index (CI), where CI < 1, CI = 1, and CI > 1 indicate synergism, additive effect, and antagonism, respectively. The CI and the dose reduction index (DRI) were estimated with the CalcuSyn v.2.0 software (Biosoft, Cambridge, UK). Furthermore, the synergistic, additive, and antagonistic effect of the drug combination was mapped with the Loewe additivity model, using the Combenefit software v.2.021 (http://sourceforge.net/projects/combenefit/).

#### Apoptosis assay

To quantify the extent of apoptosis, 3 × 10^5^ Toledo, OCI-LY3 and SU-DHL10 cells were treated for 48 h with mVNR, mETO, tafasitamab and their concomitant combination at the experimental IC_50_ or with vehicle alone as control. At the end of the experiment, control and treated cells were analyzed using the Cell Death Detection ELISA Plus Kit (catalogue #11,774,425,001, Roche, Basel, Switzerland) as per the manufacturer’s instructions. The optical density was measured using a Multiskan Spectrum microplate reader (Thermo Labsystems, Milan, Italy) set to a wavelength of 405 nm (with a wavelength of 490 nm correction). The internal negative control (absence of apoptotic signal) was provided by the ELISA kit. All the absorbance values were plotted as a percentage of apoptosis relative to IgG1-treated cells which were labelled as 100%.

#### Luminex analysis of protein phosphorylation in DLBCL cell lysates

DLBCL cells (5 × 10^4^) were plated and treated with mVNR, mETO and tafasitamab alone, or in concomitant combination at the experimental cytotoxic IC_50_ and vehicle alone for 2 h (three replicates per sample). At the end of the experiment, the cells were lysed at 4 °C with Milliplex® lysis buffer (catalogue #43–040) supplemented with protease inhibitors and then the samples filtered with Ultrafree®-MC centrifugal filter devices with microporous membranes from MerckMillipore (Merck KGaA, Darmstadt, Germany). Twenty-five μL of filtered lysate were diluted in assay buffer (1:2 v:v, respectively) (catalogue #43–041) and then a 25 μL sample of the solution was evaluated by Luminex using the MILLIPLEX® Akt/mTOR Phosphoprotein 11-plex Magnetic Bead kit (catalogue #48-611MAG kit) purchased from MerckMillipore. The samples were loaded into a 96 well-plate supplied by the kit. In each well an equal volume of a premix of 11 Luminex beads was added, followed by incubation overnight at 4 °C. The beads were subsequently washed and incubated with 25 μL of secondary biotinylated detection antibody for 1 h at room temperature, according to the manufacturer’s protocol. The samples were analyzed by FlexMap3D instrument (MerckMillipore) with xPONENT® software (MerckMillipore) following the manufacturer’s settings. Results were reported as the percentage of the phosphorylated protein versus 100% of phosphoprotein in IgG1-treated cells.

#### UPLC-HRMS analysis of vinorelbine and etoposide intracellular concentrations in DLBCL cells

Intracellular levels of vinorelbine were performed using an ultra-high performance liquid chromatography-high resolution mass spectrometry (UPLC-HRMS) as previously described by our group^21^. The same method was used and appropriately validated to determine the intracellular levels of etoposide. Validation experiments of the developed method included standard parameters including selectivity, calibration curve, lower limit of quantification, carry-over, accuracy, precision, recovery, dilution integrity, and stability as described in the European Medicine Agency (EMA) guidelines [https://www.ema.europa.eu/en/documents/scientific-guideline/guideline-bioanalytical-method-validation_en.pdf]. Briefly, human DLBCL cells (1 × 10^6^) were incubated with tafasitamab (100 nM), vinorelbine (500 nM), and etoposide (5 or 10 μM) and their combinations in RPMI-1640 for 4 and 24 h. After incubation, the cells were rinsed twice with PBS pH 5 (catalogue #D8537) at 4 °C and stored in 50 μL of methanol (catalogue #34,860). Before analysis, 100 µL of internal standard solution (vinblastine and etoposide-d3) was added. The lymphoma cells then were lysed by ultrasound (3 times for 10 s) and centrifuged at 12,000 rpm for 10 min. 100 µL of the supernatant was transferred into a vial and 10 µL was injected into the LC-HRMS system. Results were expressed as the ng/ml drug concentration and normalized to 10^5^ cells.

#### ABCB1, ABCG2 and c-MYC gene expression

Vinorelbine and etoposide are recognized substrates of two multi-drug resistant (MDR) transporters: human ATP-binding cassette sub-family B member 1 (ABCB1) and ATP-binding cassette sub-family G member 2 (ABCG2)^22^. The c-MYC, oncogene encodes a family of transcription factors that are among the most commonly activated oncoproteins in human cancers. The suppression of MYC signaling can result in sustained tumor regression^23^. To evaluate the modulation of the mRNA expression of the genes encoding for human *ABCB1*, *ABCG2* and *c-MYC*, SU-DHL10 cells were treated for 24 h with VNR and tafasitamab alone at concentrations corresponding to 500 nM and 100 nM, respectively, or to their metronomic IC_50_s, or in the concurrent combination or with IgG1 isotype control alone. An Applied Biosystems QuantStudio3 qPCR system (Applied Biosystems, Waltham, MA, USA) was used to carry out the quantitative RT-PCR. The validated primer was obtained from Applied Biosystems (*ABCB1*, Assay ID Hs01067802_m1; *ABCG2*, Assay ID Hs01053796_m1; *c-MYC*, Assay ID Hs00153408_m1). The manufacturer’s instructions were followed to set the polymerase chain reaction thermal cycling conditions. Glyceraldehyde 3-phosphate dehydrogenase (GAPDH) amplifications were used for normalization, and the ΔΔCt will be calculated; the amount of target will be defined as 2^−ΔΔCt^.

### In vivo experiments

Five-week-old Athymic Nude-Foxn1^nu^ (CD *nu/nu*) male mice, supplied by Envigo (Milan, Italy), were housed in microisolator cages on vented racks, manipulated using aseptic techniques, and were allowed unrestricted access to sterile food and water. Housing and all procedures involving animals were performed according to the protocol first approved by the Academic Organization Responsible for Animal Welfare [Organismo Preposto per il Benessere Animale (OPBA)] of the University of Pisa, in accordance with the Italian law D.lgs. 26/2014, and by the Italian Ministry of Health (Authorization No. 111/2022-PR). Each experiment employed the minimum number of mice needed to obtain statistically meaningful results. All mice were randomized just prior to initiation of treatment using = RAND() in Microsoft Excel (Microsoft Corporation, Redmond, Washington, USA). All procedures were carried out in accordance with ARRIVE guidelines.

#### Subcutaneous DLBCL xenograft models

On day 0, 5 × 10^6^ ± 5% viable Toledo (experiment #1), OCI-LY3 (experiment #2) and SU-DHL10 (experiment #3) cells/mouse were inoculated subcutaneously with Poloxamer 407 (catalogue #16,758), an inert compound that allows the formation of a solid tumor mass and at the same time has no antitumor effect^24^. Animal weights were monitored, and tumor size was measured with calipers. Tumor volume (mm^3^) was defined as follows: [(w1 × w1 × w2) × (π/6)], where w1 and w2 were the smallest and the largest tumor diameter (mm), respectively, as previously described^25^. Treatment was delivered once the tumor reached approximately 100 mm^3^, from day 6–15 after cell inoculation.

In experiment #1, the animals were randomized into four treatment groups (n = 6/group) as follows: 1) tafasitamab 10 mg/kg i.p. twice a week^4^; 2) mVNR 4 mg/kg i.p. thrice a week^26,27^; 3) simultaneous combination of mVNR and tafasitamab at each of the above doses; 4) human IgG1 isotype control 10 mg/kg i.p. twice a week as controls, for 42 days.

In experiment #2 and #3, the animals were randomized into four treatment groups (n = 6/group) as follows: 1) tafasitamab 10 mg/kg i.p. once a week; 2) mVNR 2 mg/kg i.p. thrice a week^26,27^; 3) simultaneous combination of mVNR and tafasitamab at each of the above doses; 4) human IgG1 isotype control 10 mg/kg i.p. once a week as controls, for 42 days.

Mice were euthanized using an anesthetic overdose (tiletamine/zolazepam > 100 mg/kg, i.p.) at the end of treatment period or when the weight loss exceeded 20% normal body mass, or the experimental-induced disease determined a life-threatening condition.

#### Systemic SU-DHL10 xenograft model

On day 0, 4 × 10^6^ ± 5% viable SU-DHL10 cells/mouse were inoculated through the tail vein of the nude mice. Animal weights were monitored. Survival in terms of overall survival (OS) of the treated compared to the control animals was evaluated by constructing Kaplan-Meyer curve. The animals were randomized into four treatment groups (n = 6/group) as follows: 1) tafasitamab 10 mg/kg i.p. once a week; 2) mVNR 2 mg/kg i.p. thrice a week; 3) simultaneous combination of mVNR and tafasitamab at each of the above doses; 4) human IgG1 isotype control 10 mg/kg i.p. once a week as controls, for 42 days. Mice were euthanized using an anesthetic overdose (tiletamine/zolazepam > 100 mg/kg, i.p.) when the weight loss exceeded 20% normal body mass, or the experimental-induced disease determined a life-threatening condition.

### Statistical analysis

All in vitro experiments were repeated, independently, three times with at least 9 samples for each drug concentration. The results (mean ± S.E.M.) of all the in vitro and in vivo experiments were subjected to analysis of variance between groups (ANOVA), followed by the Student–Newman–Keuls and Bonferroni post-hoc test. The level of significance was set at *P* < 0.05. Statistical analyses were performed using the GraphPad Prism software package version 9.0 (GraphPad Software, USA).

In the in vivo experiments, 6 animals (n = 6) were included in each of the groups in order to give 80% power to one-way ANOVA analysis, against a difference in tumor volumes equal or greater than 0.50*sd, with a nominal alpha error rate = 0.05. The power calculation was performed with the G*Power 3 software. The difference in overall survival among treatment profiles in vivo was assessed with the log-rank test and the Kaplan–Meier method to evaluate survival curves. The investigators responsible for data analysis were blinded to which samples/animals represented treatments and vehicle-treated controls.

## Results

### In vitro experiments

#### Tafasitamab, metronomic vinorelbine and etoposide inhibit DLBCL cell viability

First, as expected, tafasitamab did not inhibit the viability of CD19^−^ HH cells after 144 h of exposure (Supplementary Fig. S2A). Then we confirmed the absence of activity of the human IgG1 isotype control on viability of CD19^+^ Toledo, OCI-LY3 and SU-DHL10 cell lines (Supplementary Fig. S2B).

**Fig. 1:**
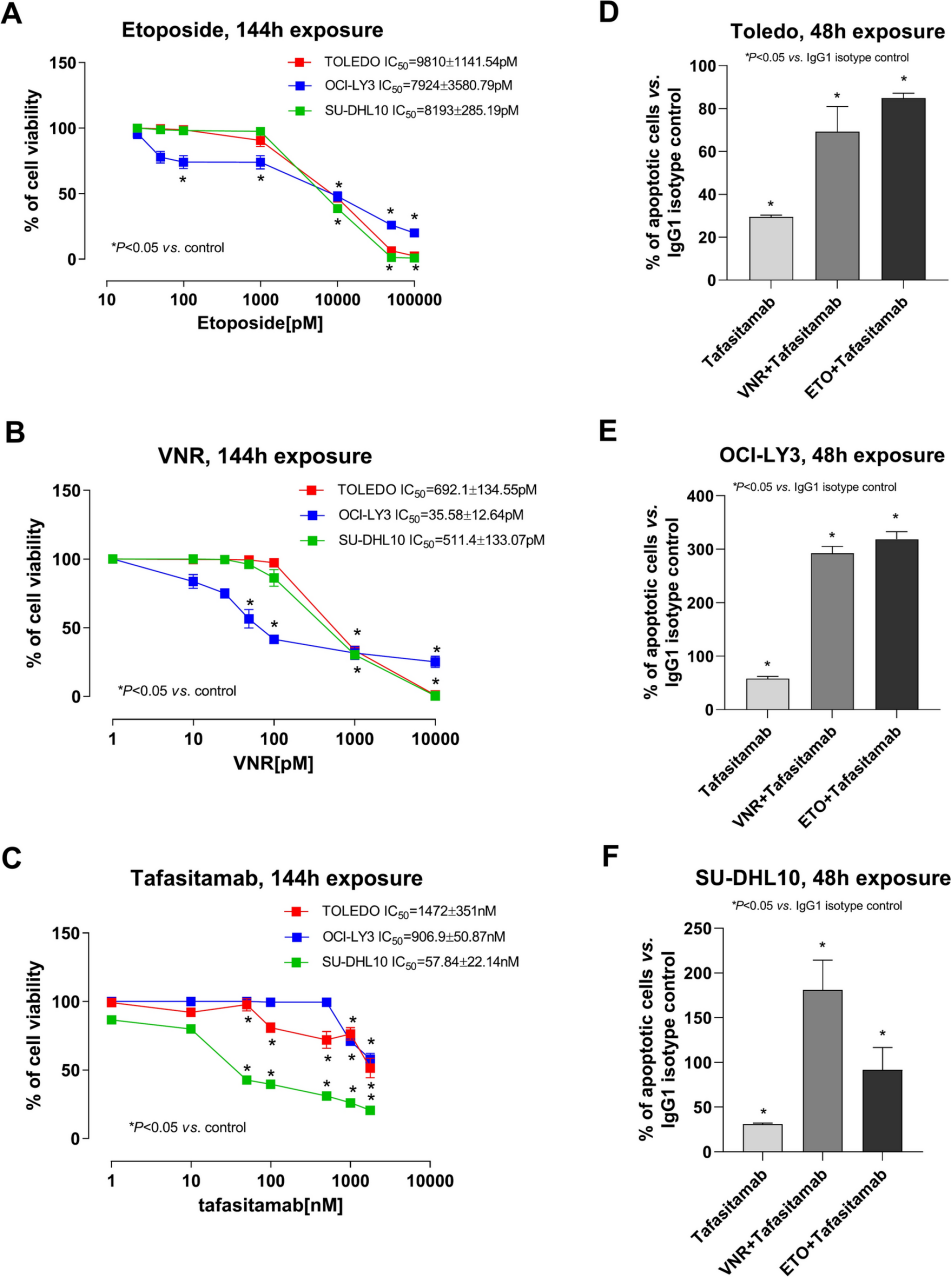
In vitro cytotoxic and pro-apoptotic activity. In vitro effect of metronomic etoposide (**A**), metronomic vinorelbine (VNR) (**B**) and tafasitamab (**C**) on viability of Toledo, OCI-LY3 and SU-DHL10 cell lines after 144 h of exposure. *Symbols and bars*, mean values ± S.E.M., respectively. Pro-apoptotic activity in Toledo (**D**), OCI-LY3 (**E**) and SU-DHL10 (**F**) cell lines treated with tafasitamab alone and in combination with metronomic vinorelbine (VNR) and metronomic etoposide (ETO) at the experimental IC_50_ for 48 h when compared to the IgG1 isotype control cells. *Columns and bars*, mean values ± S.E.M., respectively. *P < 0.05 *vs.* human IgG1 isotype controls.

Tafasitamab and both metronomic chemotherapeutic drugs, mVNR and mETO, inhibited in vitro the viability of CD19^+^ Toledo, OCI-LY3 and SU-DHL10 cell lines in a concentration-dependent manner. The 144 h mETO exposure inhibited the Toledo, OCI-LY3 and SU-DHL10 cell viability with an IC_50_ of 9.81 ± 1.14 nM, 7.92 ± 3.6 nM and 8.19 ± 0.29 nM, respectively (Fig. 1A). A higher cytotoxic effect of mVNR on Toledo, OCI-LY3 and SU-DHL10 cell lines, was found as demonstrated by the calculated IC_50_s of 692.1 ± 134.55 pM, 35.58 ± 12.64 pM and 511.4 ± 133.07 pM, respectively (Fig. 1B). The 144 h tafasitamab treatment inhibited the Toledo, OCI-LY3 and SU-DHL10 cell viability with an IC_50_ of 1,472 ± 351 nM, 906.9 ± 50.87 nM and 57.84 ± 22.14 nM, respectively (Fig. 1C).

A significant pro-apoptotic activity was found in Toledo (Fig. 1D), OCI-LY3 (Fig. 1E) and SU-DHL10 (Fig. 1F) cell lines treated with tafasitamab alone for 48 h, at a concentration corresponding to its experimental IC_50_, compared with the IgG1 isotype control, whereas the combination of tafasitamab with mVNR and mETO further enhanced mAb-induced apoptosis.

#### Synergistic effect of tafasitamab combined with metronomic vinorelbine and etoposide on DLBCL cell viability

**Fig. 2:**
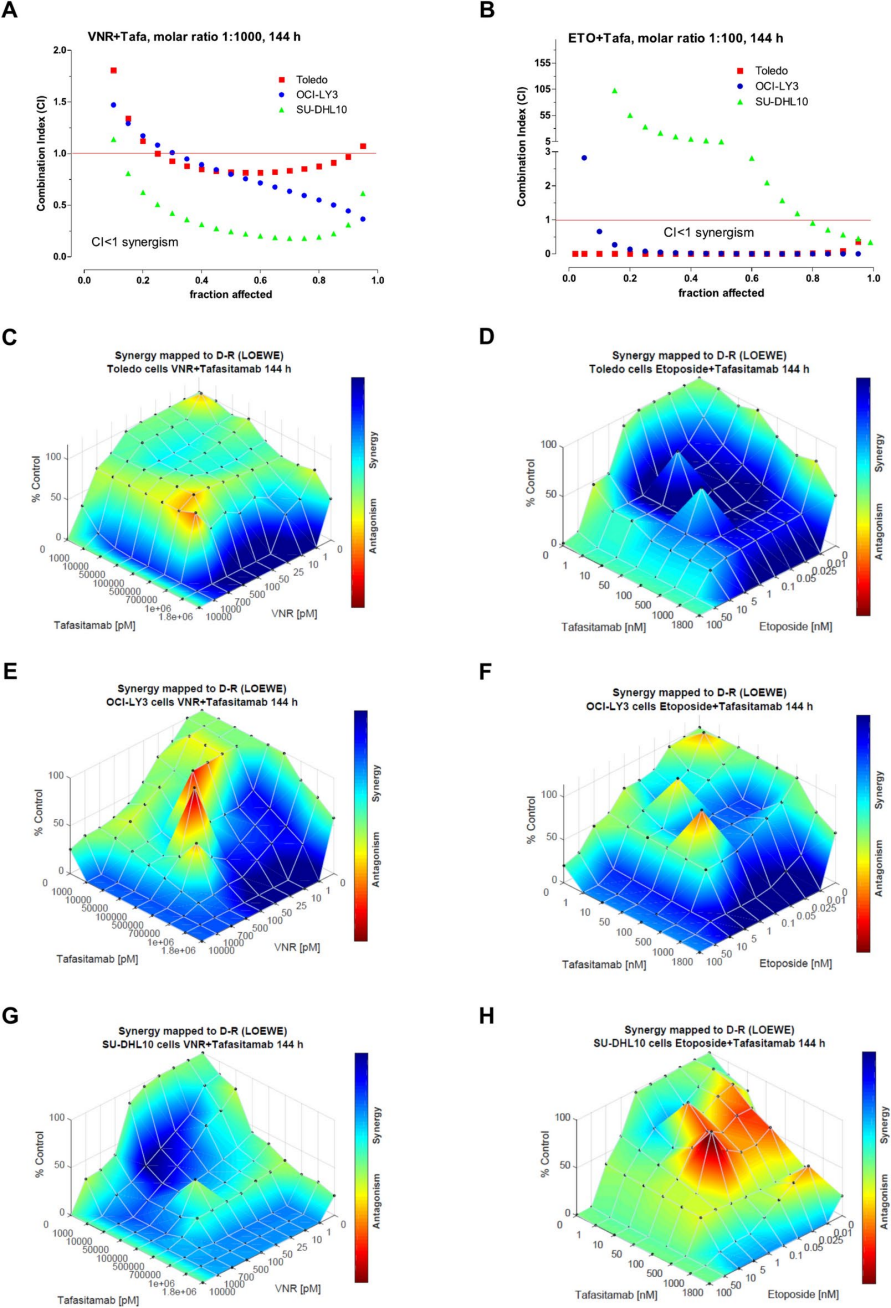
Synergistic effect of tafasitamab plus metronomic chemotherapy. Combination index (CI)-fraction affected plot of 144 h metronomic vinorelbine (VNR) + tafasitamab (TAFA) (**A**) and metronomic etoposide (ETO) + tafasitamab (**B**) concomitant combination in Toledo, OCI-LY3 and SU-DHL10 cells. CI < 1, CI = 1 and CI > 1 indicate synergism, additive effect and antagonism, respectively. Three-dimensional landscape of the dose matrix of combination responses on the Loewe model, of 144 h metronomic vinorelbine (VNR) + tafasitamab (**C**, **E**, **G**) and metronomic etoposide (ETO) + tafasitamab (**D**, **F**, **H**) concomitant combination in Toledo (**C**,** D**), OCI-LY3 (**E**, **F**) and SU-DHL10 (**G**,** H**) cells. Blue and red colour reflect evidence of synergy and antagonism, respectively. The model supported synergy of the combination in reducing cells viability. Cell viability was plotted as % human IgG1 isotype control.

In all DLBCL cell lines, simultaneous exposure to tafasitamab plus mVNR or mETO showed a marked synergism, with CI < 1 from the 30% level of cell viability inhibition (Fig. 2A) or for all fractions of affected (Fa) cells (Fig. 2B). The only exception was the tafasitamab plus mETO combination on SU-DHL10 cells that was mainly antagonistic, with CI > 1 for all the affected cell fractions (Fig. 2B). Synergism corresponding to CI < 1 always yielded a favorable DRI (> 1) for both drugs of the combination (Table 1), except for tafasitamab and mETO in SU-DHL10 cells with some DRIs < 1 (Table 1). The Loewe analysis of synergism (Fig. 2C, D, E, F, G) confirmed the findings obtained with the Chou method, but also showed areas of additivity effect (light blue and green colors), mainly for low percentages of cell viability inhibition. The Loewe analysis validated the antagonism for tafasitamab and mETO in SU-DHL10 cell line (Fig. 2H). In OCI-LY3 and SU-DHL10 cells, the triple concomitant combination of tafasitamab plus mVNR plus mETO demonstrated synergism, with CI < 1 for almost all affected cell fractions (Supplementary Fig. S3) and DRI values greater than 1 for all three drugs of the combination (Supplementary Table S1), while it was frankly antagonistic in Toledo cells (Supplementary Fig. S3) with DRI values mainly less than 1 for both vinorelbine and etoposide (Supplementary Table S1).

**Table 1:** Dose Reduction Index values for the drugs combination at 30%, 50%, 70% and 90% levels of inhibition of Toledo, OCI-LY3 and SU-DHL10 cell viability after 144 h of metronomic vinorelbine (VNR) or etoposide (ETO) plus tafasitamab (TAFA) concomitant combination treatment.

Fraction affected	DRI values					
	*Toledo*		*OCI-LY3*		*SU-DHL10*	
	VNR	TAFA	VNR	TAFA	VNR	TAFA
30%	1.6	3.1	1.09	14.7	90.5	2.4
50%	1.4	7.5	1.4	15.4	37.2	4.5
70%	1.3	18.2	1.8	16.2	15.3	8.6
90%	1.04	74.4	2.7	17.5	3.7	23.7

	ETO	TAFA	ETO	TAFA	ETO	TAFA
30%	4079.6	7463.2	21.2	766.2	1.4	0.05
50%	801.7	3712.6	110.6	842.7	1.6	0.22
70%	157.5	1846.9	577.3	926.9	1.7	0.99
90%	11.8	607.2	8028.7	1078.6	2.1	11.21

#### Tafasitamab plus metronomic vinorelbine significantly inhibit phosphorylation of GSK3α/β, mTOR, p70S6K, RPS6 and TSC2 proteins in DLBCL cells

**Fig. 3:**
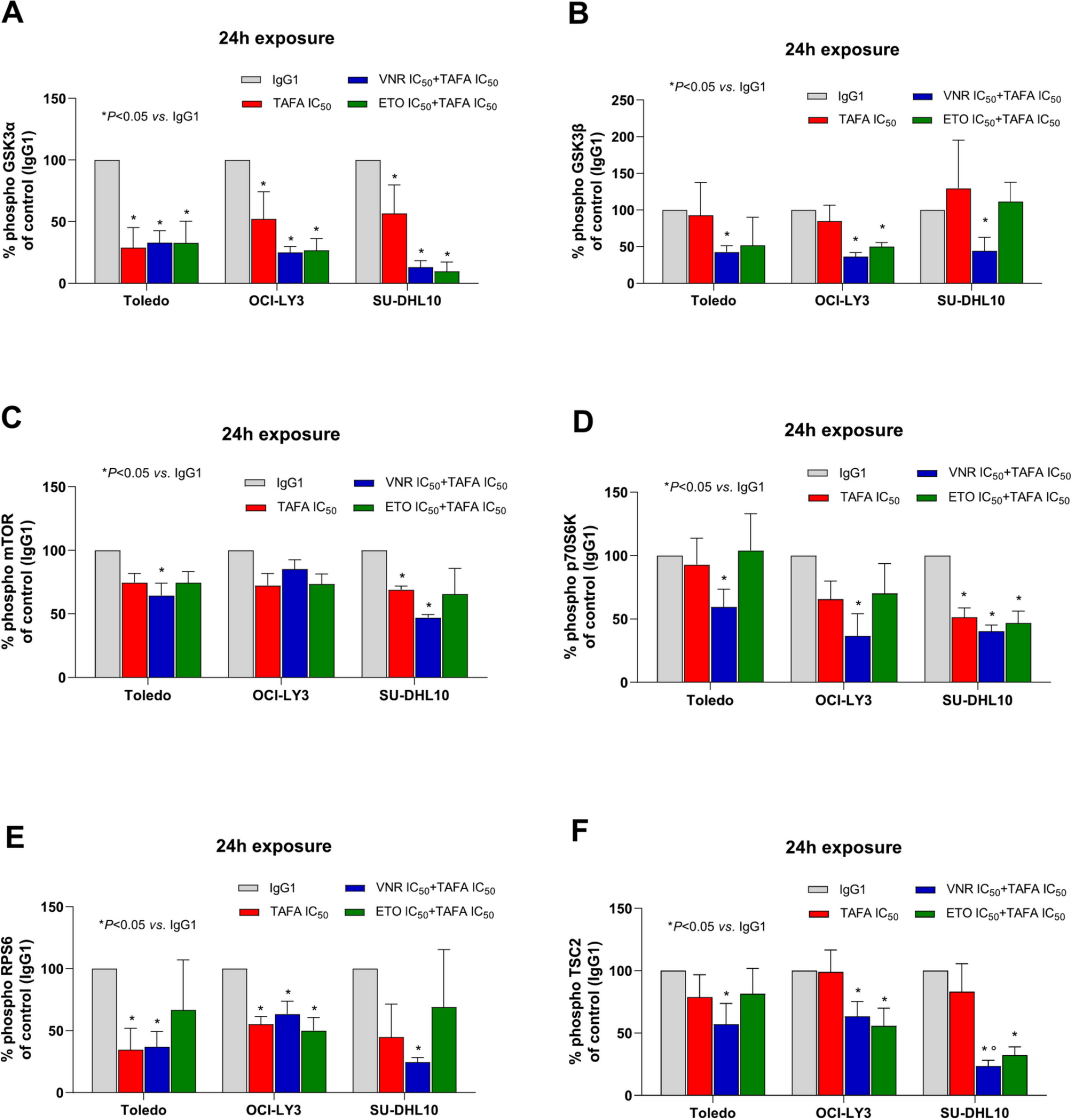
Tafasitamab plus metronomic vinorelbine significantly inhibit phosphorylation of the Akt/mTOR signaling pathway. Luminex analysis of phospho GSK3α (**A**), GSK3β (**B**), mTOR (**C**), p70S6K (**D**), RPS6 (**E**) and TSC2 (**F**) in Toledo, OCI-LY3 and SU-DHL10 cells treated with tafasitamab (TAFA) alone or in concomitant combination with vinorelbine (VNR) and etoposide (ETO) for 24 h at their experimental cytotoxic IC_50_s. Results were reported as the percentage of the phosphorylated protein *vs*. 100% of phosphorylated proteins in IgG1-treated cells. *Columns and bars*, mean values ± S.E.M., respectively. *P < 0.05 *vs*. human IgG1 isotype controls; *°P* < 0.05 for TAFA *vs*. VNR + TAFA.

To deeply investigate other molecular mechanism underlying the pharmacological activity exhibited by our new schedules, the ability of tafasitamab, alone or in combination with mVNR and mETO, to inhibit the phosphorylation of some proteins involved in the Akt/mTOR cell signaling pathway was investigated by Luminex analysis on cell lysates. The tafasitamab plus mVNR combination showed the most promising results. In fact, it significantly inhibited GSK3α (Fig. 3A), GSK3β (Fig. 3B), mTOR (Fig. 3C), p70S6K (Fig. 3D), RPS6 (Fig. 3E) and TSC2 (Fig. 3F) phosphorylation after 24 h, at concentrations corresponding to the experimental IC_50_, compared with IgG1 control, in all DLBCL cells, except for a lower, not significant, reduction of phospho-mTOR levels in OCI-LY3 cells (Fig. 3C). The observed inhibition of phosphorylation was further enhanced compared with tafasitamab alone. Interestingly, single tafasitamab treatment also significantly reduced levels of phospho-GSK3α in all DLBCL cells (Fig. 3A), phospho-mTOR and phospho-p70S6K in SU-DHL10 cells (Fig. 3C), phospho-RPS6 in Toledo and OCI-LY3 cells (Fig. 3E). Finally, the combination of tafasitamab and mETO markedly inhibited the phosphorylation of GSK3α protein in all cell lines (Fig. 3A), of GSK3β and RPS6 proteins in OCI-LY3 cells (Fig. 3B and E, respectively), of p70S6K and TSC2 proteins in SU-DHL10 cells (Fig. 3D and F, respectively).

#### Tafasitamab increases intracellular vinorelbine and etoposide concentrations in DLBCL cells

**Fig. 4:**
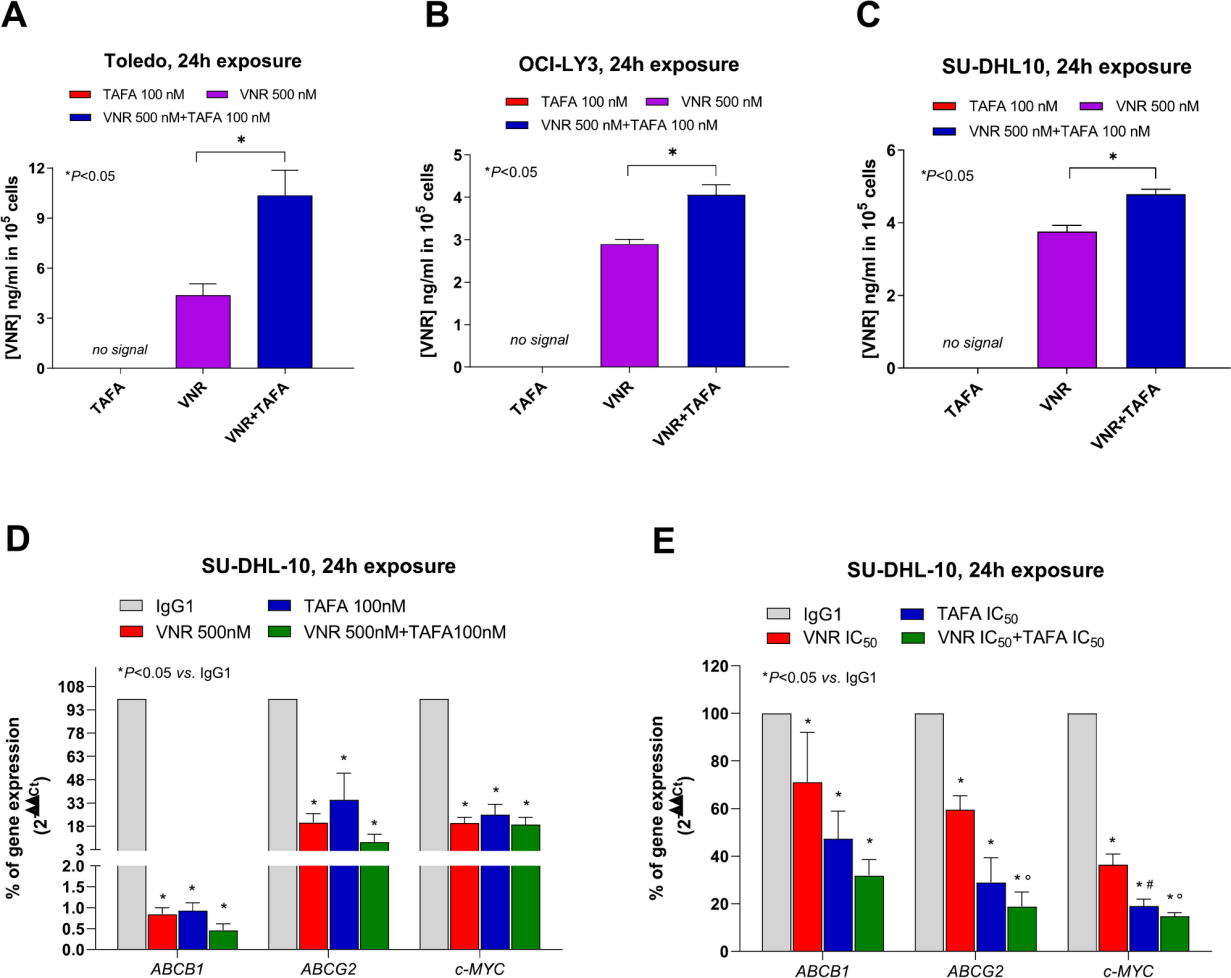
Tafasitamab increases vinorelbine intracellular concentration and their combination inhibits ABCB1, ABCG2 and c-MYC gene expression. Intracellular accumulation of vinorelbine (VNR) in Toledo (**A**), OCI-LY3 (**B**) and SU-DHL10 (**C**) cells after 24 h exposure to VNR 500 nM alone and in combination with tafasitamab (TAFA) 100 nM. *Columns and bars* indicate the mean values ± S.E.M. expressed as the ng/ml of VNR and normalized to 10^5^ cells. *P < 0.05 with respect to VNR alone. *ABCB1*, *ABCG2* and *c-MYC* gene expression (2^−ΔΔct^) levels in SU-DHL10 cells exposed to vinorelbine (VNR), tafasitamab (TAFA) and their combination, at concentrations corresponding to (**D**) 500 nM and 100 nM, respectively, or to (**E**) the metronomic IC_50_s, or with human IgG1 isotype control alone for 24 h. Data are expressed as percentage of vehicle-treated cells. *Columns and bars*, mean values ± S.E.M., respectively. *P < 0.05 *vs*. human IgG1 isotype controls; *°P* < 0.05 for VNR *vs*. VNR + TAFA; ^*#*^*P* < 0.05 for VNR *vs.* TAFA.

To explore the synergistic effects observed with the tafasitamab plus mVNR and mETO combinations, a variation of the intracellular VNR and ETO level was considered. A significant increase in VNR intracellular concentrations was found in all DLBCL cell lines after 24 h of treatment with the combination of VNR plus tafasitamab compared to cells treated with VNR alone (Fig. 4A, B and C). Interestingly, VNR intracellular concentrations started to increase already after 4 h of combined treatment, but without significant differences (Supplementary Fig. S4A, S4B and S4C). In Toledo cells, ETO intracellular concentrations significantly increased after 4 h (Supplementary Fig. S5A, S5B) and 24 h (Supplementary Fig. S6A, S6B) of treatment with ETO plus tafasitamab compared to cells treated with ETO alone. In OCI-LY3 cells (Supplementary Fig. S5C, S5D, S6C, S6D), treatment with ETO 5 μM and 10 μM plus tafasitamab 100 nM resulted in elevated ETO intracellular concentrations after at least 24 h of exposure (Supplementary Fig. S6C and S6D). However, the increase was significant only for the combination with the lower ETO dosage (Supplementary Fig. S6C). In SU-DHL10 cells, no significant differences in ETO intracellular concentrations were observed at different time points of combined treatment with ETO plus tafasitamab 100 nM, consistently with the antagonistic effect observed (Supplementary Fig. S5E, S5F, S6E, S6F).

#### Gene expression changes induced by vinorelbine, tafasitamab and their combination

The modulation of *ABCG2*, *ABCB1*, and *c-MYC* gene expressions was evaluated. The *ABCG2* and *ABCB1* genes transporter mRNA expression was suppressed in SU-DHL10 cells treated for 24 h with VNR 500 nM, tafasitamab 100 nM, and, in particular, with their concomitant combination. In SU-DHL10 cells, the *ABCB1* gene expression was dramatically decreased (Fig. 4D) after the tafasitamab, VNR and, in greater extent, in their combination treatment. Similarly, *ABCG2* mRNA expression significantly and highly decreased following treatment in the concomitant combination of tafasitamab with VNR (Fig. 4D). In SU-DHL10 cells, also *c-MYC* mRNA expression was reduced in the tafasitamab and VNR alone treatment, and in the concomitant combination with VNR (Fig. 4D). Interestingly, a significant inhibition of *ABCB1*, *ABCG2* and *c-MYC* gene expression was observed in SU-DHL10 exposed for 24 h to much lower concentrations of VNR, tafasitamab, and their concurrent combination corresponding to the experimental IC_50_s obtained in cytotoxicity assays (Fig. 4E). The combined treatment at IC_50_ strikingly decreased *ABCB1*, *ABCG2* and *c-MYC* mRNA expression compared to single treatment (Fig. 4E).

### In vivo experiments

#### Subcutaneous DLBCL xenograft models

**Fig. 5:**
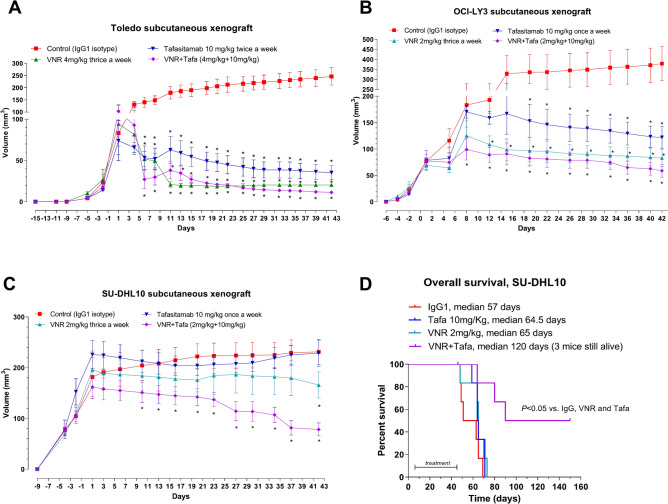
In vivo antitumor activity. Antitumor effect (**A**) of tafasitamab (TAFA) 10 mg/kg i.p. twice a week, metronomic vinorelbine (mVNR) 4 mg/kg i.p. thrice a week, concomitant combination of tafasitamab and mVNR at each of the above doses, and human IgG1 isotype control i.p. twice weekly as controls for 42 days, on Toledo tumors subcutaneously xenotransplanted in CD *nu/nu* mice. Antitumor effect (**B**) of tafasitamab (TAFA) 10 mg/kg i.p. once a week, metronomic vinorelbine (mVNR) 2 mg/kg i.p. thrice a week, concomitant combination of tafasitamab and mVNR at each of the above doses, and human IgG1 isotype control i.p. once a week as controls for 42 days, on OCI-LY3 tumors subcutaneously xenotransplanted in CD *nu/nu* mice. Antitumor effect (**C**) of tafasitamab (TAFA) 10 mg/kg i.p. once a week, metronomic vinorelbine (mVNR) 2 mg/kg i.p. thrice a week, simultaneous combination of tafasitamab and mVNR at each of the above doses, and human IgG1 isotype control i.p. once a week as controls for 42 days, on SU-DHL10 tumors subcutaneously xenotransplanted in CD *nu/nu* mice. Overall survival (**D**) of CD *nu/nu* mice intravenously inoculated with SU-DHL10 cells and treated with tafasitamab (TAFA) 10 mg/kg i.p. once a week, metronomic vinorelbine (mVNR) 2 mg/kg i.p. thrice a week, simultaneous combination of tafasitamab and mVNR at each of the above doses, and human IgG1 isotype control i.p. once weekly as controls for 42 days (treatment period). *P < 0.05 with respect to IgG1 isotype controls. *Symbols and bars*, mean ± S.E.M.

In experiment #1, we established a subcutaneous Toledo xenograft in CD *nu/nu* mice. Both mVNR (4 mg/kg thrice a week) and tafasitamab (10 mg/kg twice a week) monotherapies significantly inhibited tumor growth compared to control animals (Fig. 5A and Supplementary Fig. S7A). The concomitant combination of tafasitamab and mVNR rapidly reduced the subcutaneous tumor volumes, and this was evident from 4 to 6 days after the start of therapy (Fig. 5A and Supplementary Fig. S7A). By the end of the 42 days of treatment, the combined schedule was the therapeutic approach with the maximum effect on tumor volumes. The combination significantly inhibited tumor growth without producing excessive toxicity although the mVNR alone group required saline integrations to recover the weight loss (Supplementary Fig. S8A).

Given the good results obtained from experiment #1, we decide to adapt the dosage and time of administration of the drugs in the next experiments, mainly to further minimize the appearance of VNR toxicity and, at the same time, maximize the antitumor activity.

In experiment #2, we established a subcutaneous OCI-LY3 xenograft in CD *nu/nu* mice. Both mVNR (2 mg/kg thrice a week) and tafasitamab (10 mg/kg once a week) monotherapies and concomitant combination significantly inhibited tumor growth compared to controls (Fig. 5B and Supplementary Fig. S7B), with no evidence of toxicity (Supplementary Fig. S8B). Also in this case, the halving of both drug doses in the simultaneous combination of tafasitamab and mVNR resulted the more effective treatment on subcutaneous tumors.

In experiment #3, we established a subcutaneous SU-DHL10 xenograft in CD *nu/nu* mice. Mice did not benefit from tafasitamab (10 mg/kg once a week) monotherapy compared to controls. However, the combination of mVNR (2 mg/kg thrice a week) and tafasitamab (10 mg/kg once a week) resulted more effective in reducing tumor volume compared with single drug treatments (Fig. 5C and Supplementary Fig. S7C), together with a favorable toxicity profile (Supplementary Fig. S8C).

#### Systemic SU-DHL10 xenograft model

To establish a systemic DLBCL xenograft model, the most aggressive cell line SU-DHL10 was inoculated through the tail of CD *nu/nu*. Single treatments with mVNR (2 mg/kg thrice a week) and tafasitamab (10 mg/kg once a week) showed only a not significant survival advantage (median survival 65 and 64.5 days, respectively) versus IgG1control mice (57 days) affected by systemic lymphoma (Fig. 5D), confirming the data of the previous in vivo experiments from the subcutaneous model. Instead, the mVNR and tafasitamab concomitant combination produced a greater survival result than both monotherapies, and resulted in a significant higher median overall survival of 120 days (*P* < 0.05 *vs*. IgG1, VNR, and tafasitamab) (Fig. 5D). The toxicity during the treatment period was absent for all groups and the weights of the mice slowly increased (Supplementary Fig. S8D).

## Discussion

In the current study, we investigated the non-immune-mediated preclinical activity of tafasitamab alone and its combination with the microtubule-targeting drug vinorelbine or the topoisomerase II inhibitor etoposide, administered in a metronomic manner, in human DLBCL cells both in vitro and in vivo.

Our findings show that tafasitamab monotherapy in CD19^+^ DLBCL cells has a direct cytotoxic effect at concentrations compatible with plasma levels in humans after drug administration^28^. This efficacy was also maintained in the in vivo subcutaneous DLBCL model in particular when the antibody was administered twice a week. The concomitant combination of these drugs, also at lower concentrations and doses, was highly synergistic on DLBCL cells and on both the in vivo DLBCL models, the subcutaneous and systemic ones, significantly increasing the mice survival.

Preclinical studies investigating the effects of the combination of anti-CD19 mAbs to metronomic chemotherapeutic agents are lacking. However, it has been demonstrated that HD37, an anti-CD19 antibody, strongly enhanced the in vitro cytotoxicity of daunorubicin and vincristine in three pre-B ALL cell lines^29^. Moreover, treatment of SCID/ALL mice with HD37 plus vincristine significantly extended their mean survival time compared to either chemotherapeutics alone. Indeed, 40% of the mice receiving the HD37 plus vincristine combination survived^29^.

Upon BCR activation, CD19 amplifies BCR-induced signaling essential for B cell expansion by enrolling and activating PI3K and downstream Akt kinases^30^. This binding can also suppress the growth of several lymphoma cell lines by causing cell cycle arrest^31^. Indeed, our results showing the decreased expression of the oncogenic transcription factor MYC and inhibition of AKT/mTOR signaling pathway confirmed a direct cytotoxic activity of tafasitamab on SU-DHL10 cells, consistently with recently reported data following treatment with tafasitamab^32^. Moreover, we showed that VNR and tafasitamab combination, greatly suppressed the *ABCB1* and *ABCG2* gene expression, suggesting an additional mechanism of negative modulation of drug efflux transporters, thus further increasing the intracellular concentrations of active chemotherapeutic drugs when combined with tafasitamab. Noteworthy, we found that tafasitamab significantly increased VNR and ETO intracellular concentrations in DLBCL cell lines, compared to cells treated without tafasitamab, (except for the antagonist combination of tafasitamab plus ETO in SU-DHL10 cells). Studies have demonstrated that the binding of an anti-mAb, such as HD37, to the CD19 cell surface receptor can hamper the activity of the P-glycoprotein (P-gp; also known as ABCB1) pump in a MDR B-lymphoma cell line^33^. Since CD19 and P-gp are constitutively associated in MDR B-lymphoma cells, anti-CD19 therapy might chemosensitize P-gp^+^ cells by disrupting CD19/P-gp interactions, swiftly causing the translocation of P-gp into a compartment on the plasma membrane where it loses its activity^34^. Therefore, this intracellular increase of drug concentrations can be conceivably also caused by the anti-CD19 therapy-induced inactivating translocation of the P-gp efflux pump as previously described.

Aberrant activations of signaling pathways are involved in the aggressive biology of B-cell NHL^1^. As, recent studies have demonstrated that the PI3K/Akt/mTOR signaling pathway is aberrantly expressed and plays a crucial role in regulating cell proliferation and survival in lymphoid malignancies^35,36^. We investigated, by Luminex analysis on cell lysates, the inhibitory activity of tafasitamab plus mCHEMO on the phosphorylation of some proteins, involved in the Akt/mTOR cell signaling pathway. Interestingly, we demonstrated that tafasitamab, also as a single-agent, significantly reduced levels of phospho-GSK3α in all DLBCL cells, phospho-mTOR and phospho-p70S6K in SU-DHL10 cells, phospho-RPS6 in Toledo and OCI-LY3 cells. Noteworthy, we observed a significant reduction of the phosphorylation of GSK3α/β, mTOR, p70S6K, RPS6 and TSC2 proteins in all DLBCL cells after treatment with tafasitamab plus mVNR. Indeed, lymphoma cells have shown an abundant expression of GSK3α/β with respect to normal B and T lymphocytes, both at the mRNA and protein levels. Using a novel GSK3 inhibitor and through genetic knockout of GSK3α/β genes, GSK3 has been demonstrated to be functionally important for lymphoma cell proliferation and survival^37^. We also found that *c-MYC* expression was strongly decreased after the combined treatment of tafasitamab and mVNR, suggesting a further inhibitory mechanism on cell survival. The extensively recognized MYC oncogene, which regulates key cellular functions such as proliferation, is dysregulated in a number of B-cell lymphomas^38^. MYC overexpression in DLBCL may be induced by the BCR–PI3K signaling pathway^39^. Indeed, the BCR–PI3K pathway may stabilize MYC by dephosphorylating its T58 site, through the phosphorylation of GSK-3β^39^. As MYC activation can be reduced via BCR signaling downregulation, our results in SU-DHL10 cells demonstrate that tafasitamab may induce direct cytotoxicity by inhibiting the Akt/mTOR pathway, which is markedly potentiated by the addition of mVNR. Therefore, our results strongly support the synergy of tafasitamab plus mCHEMO combination in directly reducing DLBCL cell viability by both enhancing intracellular concentrations of chemotherapeutic drugs and affecting phosphorylation of most proteins involved in the Akt/mTOR signaling pathway, which play a fundamental role in controlling lymphoma cell growth and survival^35^ and downregulating the *MYC* gene expression.

In all DLBCL cell lines, mVNR proved the greatest cytotoxic activity, as highlighted by the picomolar IC_50_ values, the strongest synergistic effect in combination with tafasitamab, and the largest increase in intracellular concentrations when combined with tafasitamab. Thus, we decided to investigate the activity of mVNR in our DLBCL xenograft mouse models and verified whether or not its combination with tafasitamab is likewise synergistic in vivo. Our in vivo models were designed to highlight the direct, non-immune-mediated, effects of the combination suggesting an additional direct mechanism to the immunological ones already knew of the anti-CD19 antibody. The enhanced antitumor effect of the concomitant combination schedule of tafasitamab and mVNR was demonstrated also in the subcutaneous DLBCL models. In particular, the combination significantly reduced tumor volume in all three subcutaneous models compared to control mice. All therapies were well tolerated in the in vivo subcutaneous DLBCL models. Moreover, the doses of both mVNR and tafasitamab were reduced by 50% after the first in vivo subcutaneous experiment resulting tolerable and above all still successful in hampering tumor growth. Additionally, we evaluated the combination therapy also on a systemic DLBCL model, to the best of our knowledge an in vivo model that recapitulates features of human disseminated non-Hodgkin lymphoma in immunodeficient mice. We reported a significant increase in survival in SU-DHL10 bearing mice treated with the tafasitamab plus mVNR combination schedule compared to control mice and single treatment. Additionally, some mice showed long-term survival and no clear signs of widespread disease at the time of sacrifice.

However, our study presents some limitations. One limitation is that, in all our in vivo subcutaneous xenografts, the enlargement of control tumors in our in vivo models slows down after an initial phase of exponential growth. We used Poloxamer 407, an inert tri-block polymer of poly(ethylene oxide)–poly(propylene oxide)–poly(ethylene oxide), to allow the formation of a solid tumor mass. Indeed, if used at low concentration, the three-dimensional physical support of poloxamer enabled an initial exponential growth of the tumor masses. However, a disadvantage of poloxamer 407 is its fast degradation rate in vivo^40^. It almost completely biodegrades after about 6 weeks. Therefore, the gel structure reasonably begins to hydrolyze at 15–20 days post inoculation, probably affecting the three-dimensional development of the tumor masses, but not cell replication. However, the difference between the tumor volume of combination treatment group and controls remains extremely significant. Additionally, our findings are encouraging, but they warrant further investigations to validate and strengthen the mechanisms underlying the synergistic effect of the TAFA + mCHEMO combination in DLBCL cells, especially more deeply investigating the Akt/mTOR pathway.

In conclusion, we report the synergistic direct activity of tafasitamab in combination with mCHEMO in DLBCL cell lines*,* both in vitro and in vivo. Our in-depth analysis unveiled several molecular interactions, which explain, at least in part, the strong synergism of the combination. Since novel therapeutic strategies are urgently needed for relapsed/refractory DLBCL, these data prompt a rapid translation, into clinical trials.

## Supplementary Information

Supplementary Information 1.

Supplementary Information 2.

## Data Availability

All data generated or analyzed during this study are available upon request to Prof. Guido Bocci at the Department of Transitional Research and of New Surgical and Medical Technologies, University of Pisa, Pisa, Italy.

## Abbreviations

ABCB1: ATP-binding cassette sub-family B member 1
ABCG2: ATP-binding cassette sub-family B member 2
ADCC: Antibody-dependent cellular cytotoxicity
ADCP: Antibody-dependent cellular phagocytosis
Akt: Protein kinase B
ASCT: Autologous stem cell transplant
BCR: B-cell receptor
CTCL: Cutaneous T-cell lymphoma
DLBCL: Diffuse large B-cell lymphoma
GSK3α/β: Glycogen synthase kinase 3 α/β
mCHEMO: Metronomic chemotherapy
MDR: Multi-drug resitant
MTD: Maximum tolerated dose
mVNR: Metronomic vinorelbine
mETO: Metronomic etoposide
mTOR: Mechanistic or mammalian target of rapamycin
NHL: Non-Hodgkin lymphoma
p70S6K: Ribosomal protein S6 kinase beta-1
PI3K: Phosphoinositide 3-kinase
RPS6: Ribosomal protein S6
TAFA: Tafasitamab
TSC2: Tuberous sclerosis complex 2
