# Supplementary material for: Synergistic activity of tafasitamab and metronomic chemotherapy on diffuse large B-cell lymphoma through inhibition of the AKT/mTOR signaling pathway
Source: Sci Rep. 2025 Apr 3;15:11372. doi: 10.1038/s41598-025-95476-y (PMC11965395; doi:10.1038/s41598-025-95476-y)
Supplement: Supplementary file 1 — Supplementary Information 1. [file 41598_2025_95476_MOESM1_ESM.docx]

**Highlights**

- Tafasitamab (TAFA), an anti-CD19 antibody, with metronomic chemotherapy (mCHEMO) exerted a direct synergistic activity on DLBCL cells
- TAFA + mCHEMO significantly inhibited the Akt/mTOR cell signaling pathway
- TAFA increased the intracellular concentrations of mCHEMO in DLBCL cells
- Combination of TAFA and mCHEMO prolonged survival of mice with DLBCL, without toxicity
