# Supplementary material for: Synergistic activity of tafasitamab and metronomic chemotherapy on diffuse large B-cell lymphoma through inhibition of the AKT/mTOR signaling pathway
Source: Sci Rep. 2025 Apr 3;15:11372. doi: 10.1038/s41598-025-95476-y (PMC11965395; doi:10.1038/s41598-025-95476-y)
Supplement: Supplementary file 2 — Supplementary Information 2. [file 41598_2025_95476_MOESM2_ESM.pdf]

Supplementary figures and tables

Synergistic activity of tafasitamab and metronomic chemotherapy on diffuse large B-cell lymphoma through inhibition of the AKT/mTOR signaling pathway

[1*§]Marta Banchi, [2§]Maria Christina Cox, [3]Paola Orlandi, [1, 3]Arianna Bandini, [4]Fabio Stefanelli, [4]Silvio Chericoni, [1]Guido Bocci

[1]Department of Translational Research and of New Surgical and Medical Technologies, University of Pisa, Pisa, Italy;

[2]Hematology Unit, Fondazione Policlinico Tor Vergata, Rome, Italy;

[3]Department of Clinical and Experimental Medicine, University of Pisa, Pisa, Italy;

[4]Department of Surgical, Medical and Molecular Pathology and Critical Care Medicine, University of Pisa, Pisa, Italy

§ these authors contributed equally to this work

***Corresponding author:**

Marta Banchi, PharmD

Department of Translational Research and of New Surgical and Medical Technologies

University of Pisa, Via Roma 55, 56126 Pisa, Italy
e-mail: m.banchi@studenti.unipi.it

https://orcid.org/0000-0003-0672-2066

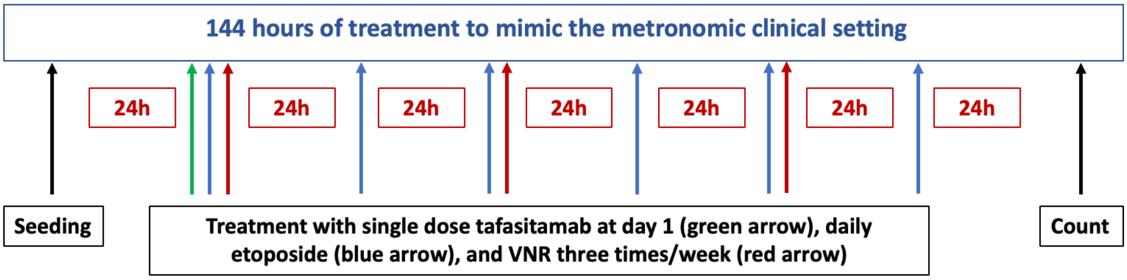

Figure S1. 144h treatment schedules of metronomic etoposide (ETO; blue arrows), metronomic vinorelbine (VNR; red arrows) and tafasitamab (green arrow), mimicking the clinical setting.

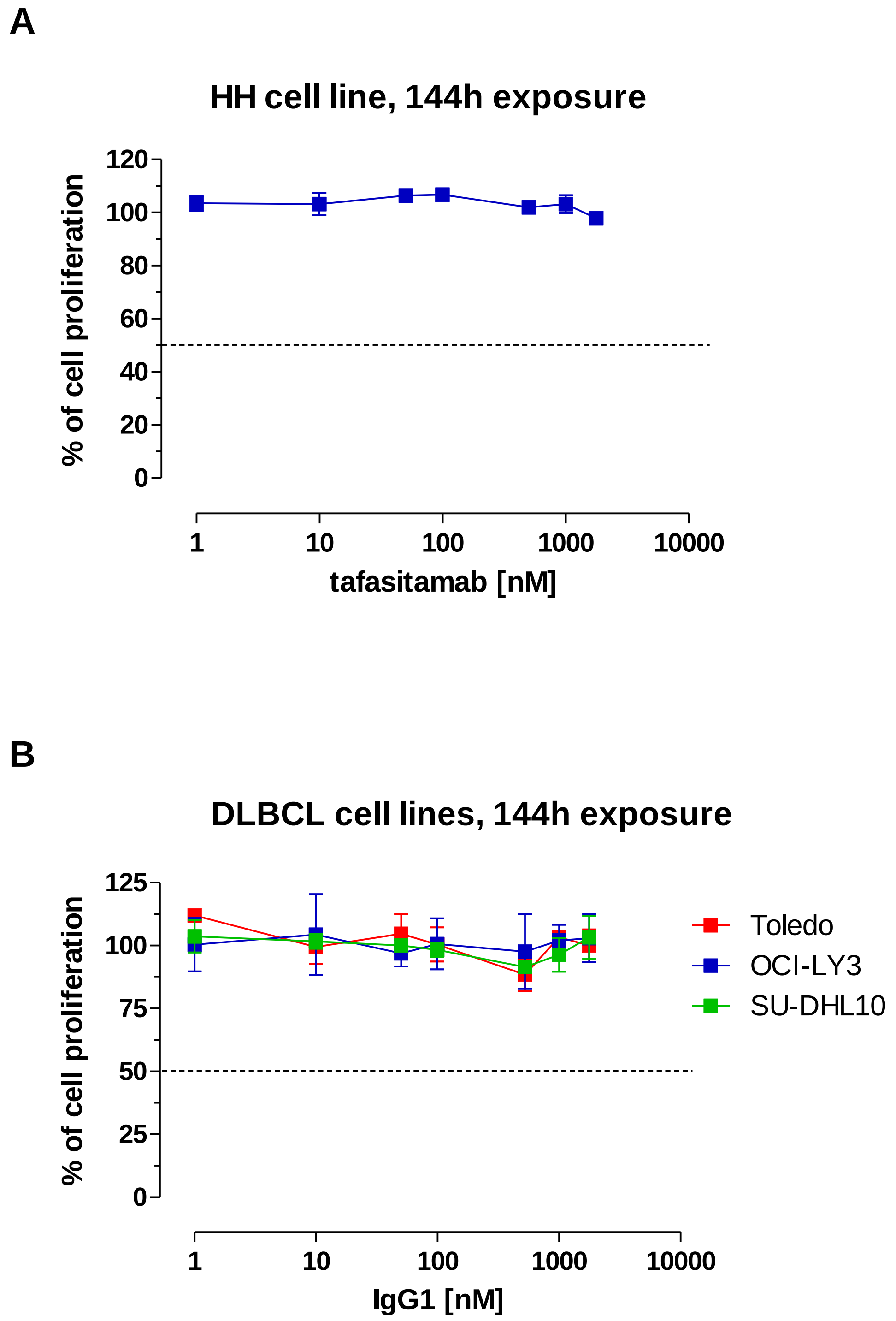

Figure S2. *In vitro* effect of (**A**) tafasitamab on proliferation of CD19$^-$ HH cell line, and of (**B**) human IgG1 isotype control on viability of Toledo, OCI-LY3 and SU-DHL10 cells after 144 h of exposure. *Symbols and bars*, mean values±S.E.M., respectively. DLBCL, diffuse large B-cell lymphoma

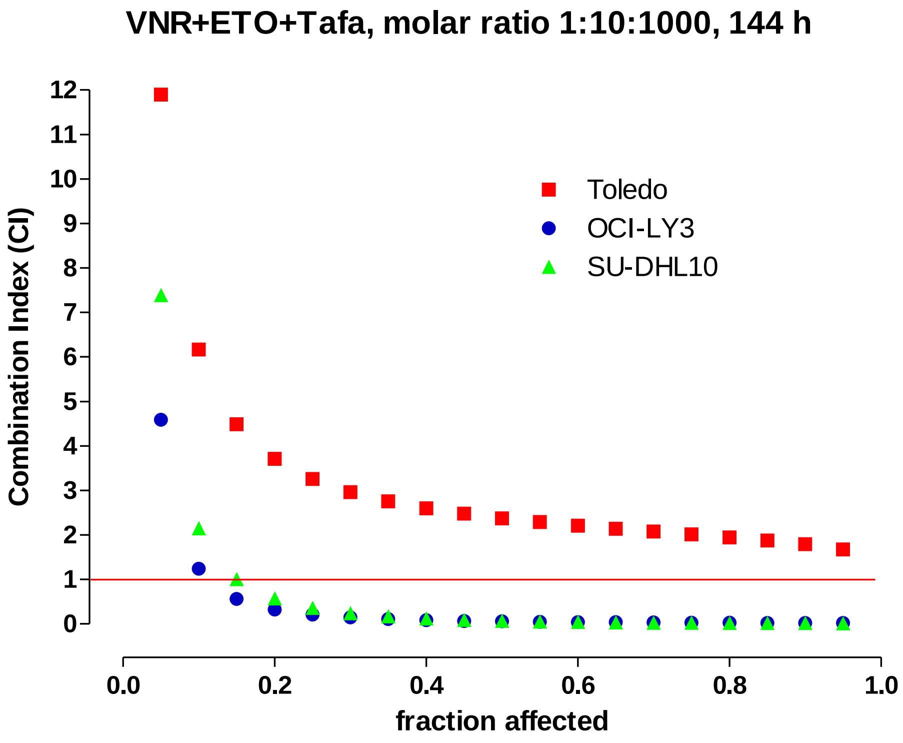

Figure S3. Combination index (CI)-fraction affected (Fa) plot of 144 metronomic vinorelbine (VNR) + metronomic etoposide (ETO) + tafasitamab (TAFA) concomitant combination in Toledo, OCI-LY3 and SU-DHL10 cells. CI<1, CI=1 and CI>1 indicate synergism, additive effect and antagonism, respectively.

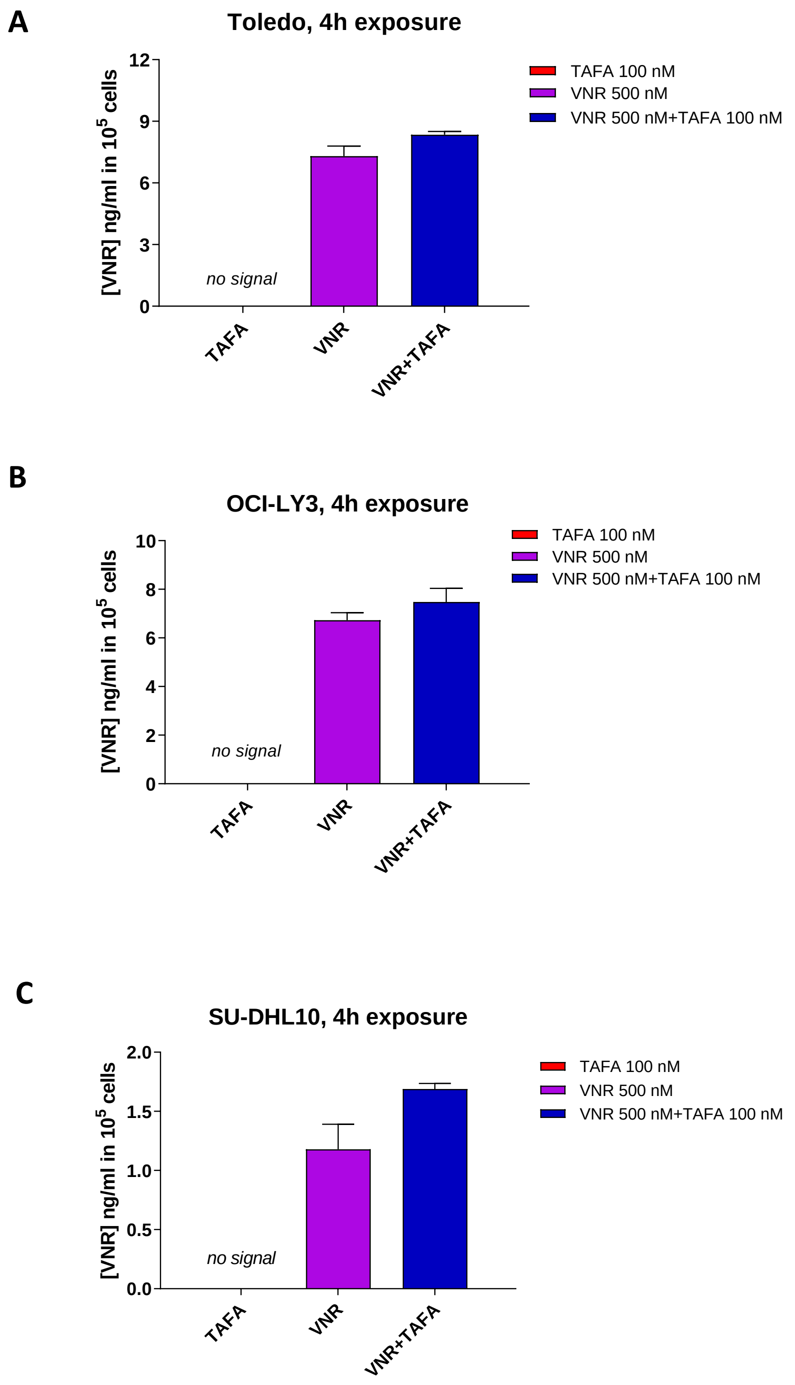

Figure S4. Intracellular accumulation of vinorelbine (VNR) in Toledo (**A**), OCI-LY3 (**B**) and SU-DHL10 (**C**) cells after 4h exposure to VNR 500nM alone and in combination with tafasitamab (TAFA) 100nM. *Columns and bars* indicate the mean percentage values ±S.E.M. *vs*. treated cells with VNR alone.

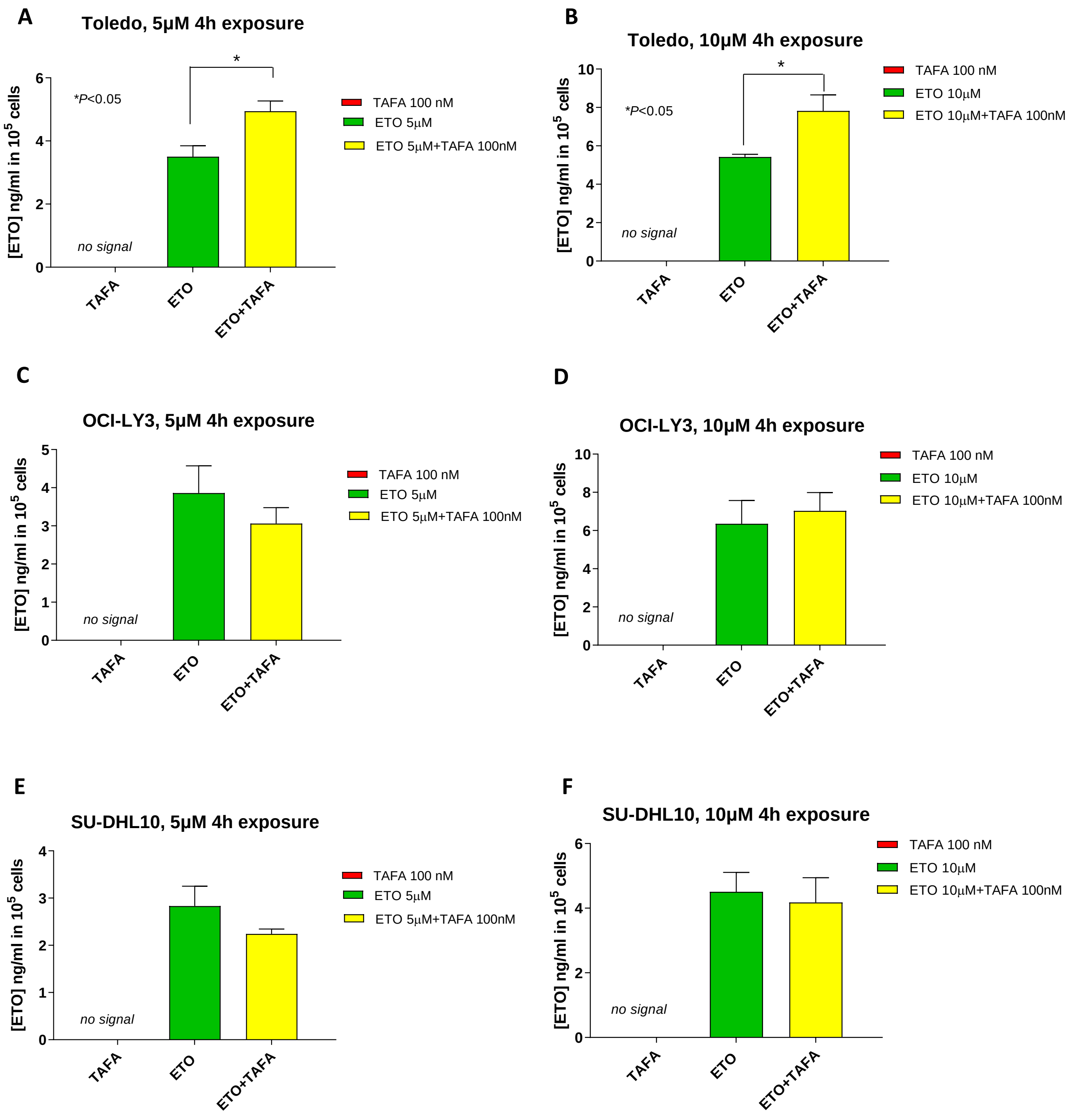

Figure S5. Intracellular accumulation of etoposide (ETO) in Toledo (**A**, **B**), OCI-LY3 (**C**, **D**) and SU-DHL10 (**E**, **F**) cells after 4h exposure to ETO 5μM (**A**, **C, E**) and 10μM (**B**, **D**, **F**) alone and in combination with tafasitamab (TAFA) 100nM. *Columns and bars* indicate the mean values ±S.E.M. expressed as the ng/ml of ETO and normalized to 10^5 cells. **P*<0.05 with respect to ETO alone.

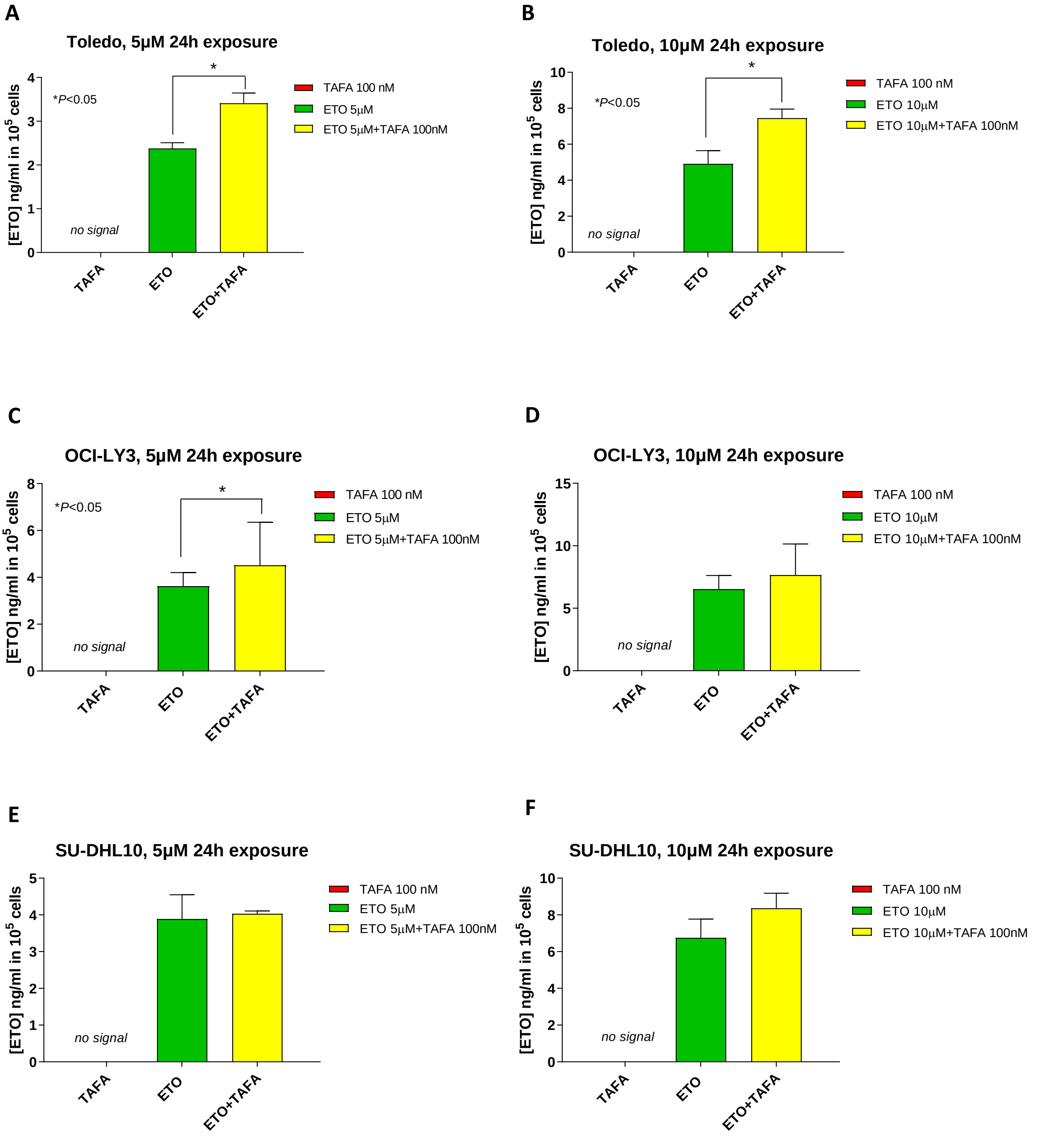

Figure S6. Intracellular accumulation of etoposide (ETO) in Toledo (**A**, **B**), OCI-LY3 (**C**, **D**) and SU-DHL10 (**E**, **F**) cells after 24h exposure to ETO 5µM (**A**, **C**, **E**) and 10µM (**B**, **D**, **F**) alone and in combination with tafasitamab (TAFA) 100nM. *Columns and bars* indicate the mean values ±S.E.M. expressed as the ng/ml of ETO and normalized to 10^5 cells. *P<0.05 with respect to ETO alone.

A

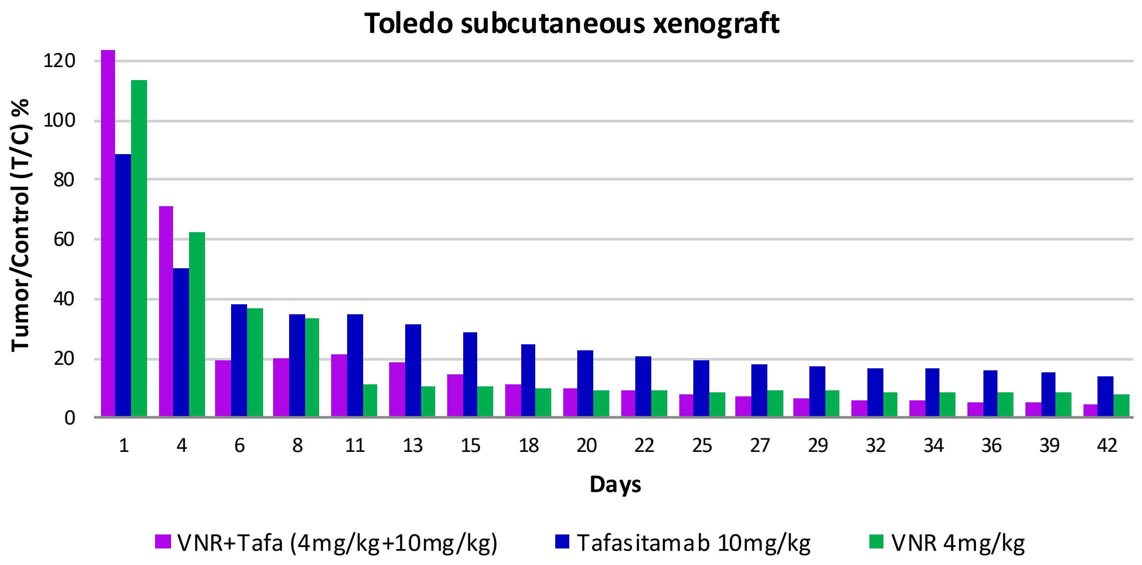

B

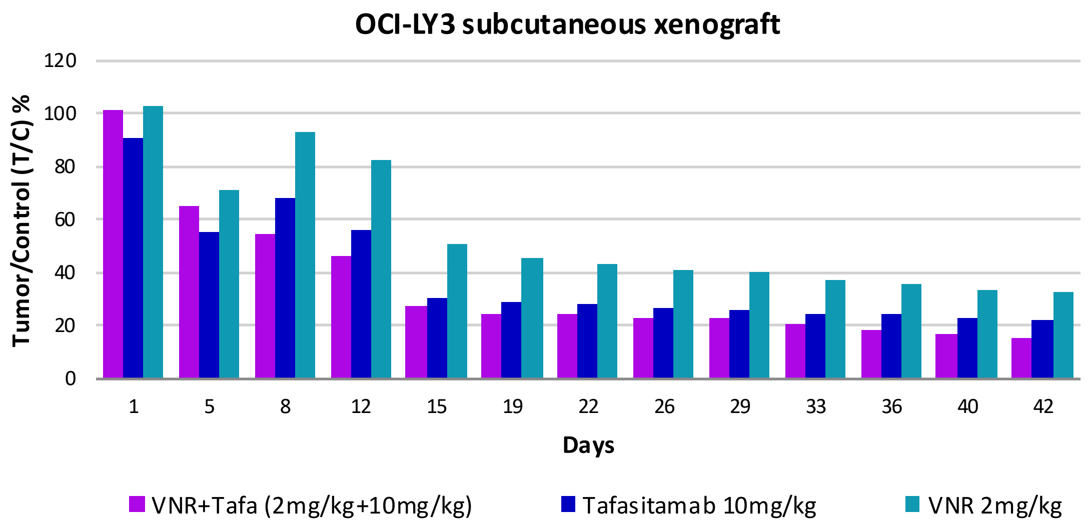

C

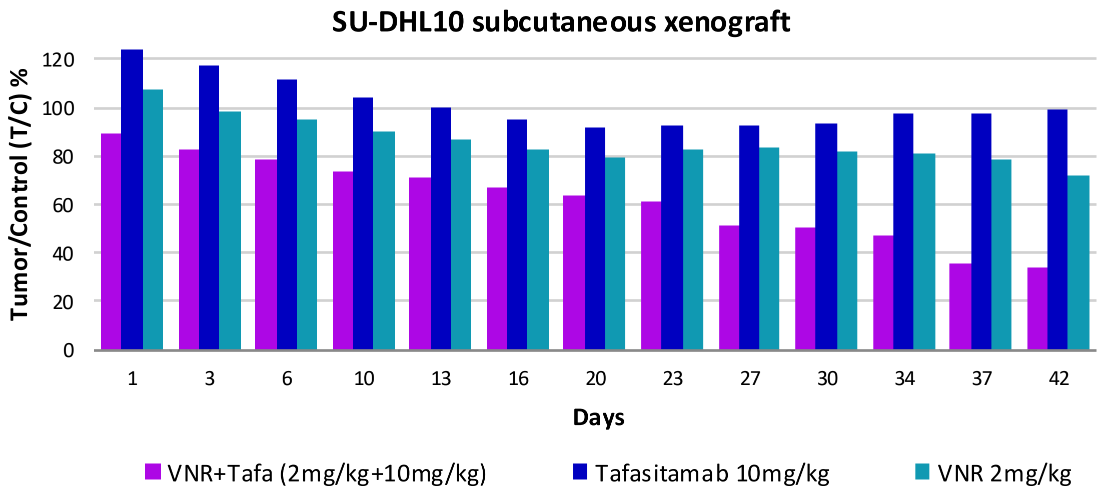

Figure S7. Treatment-to-control (T/C) ratio from tumor volumes of Toledo (**A**), OCI-LY3 (**B**) and SU-DHL10 (**C**) subcutaneous xenografts. TAFA, tafasitamab; VNR, vinorelbine.

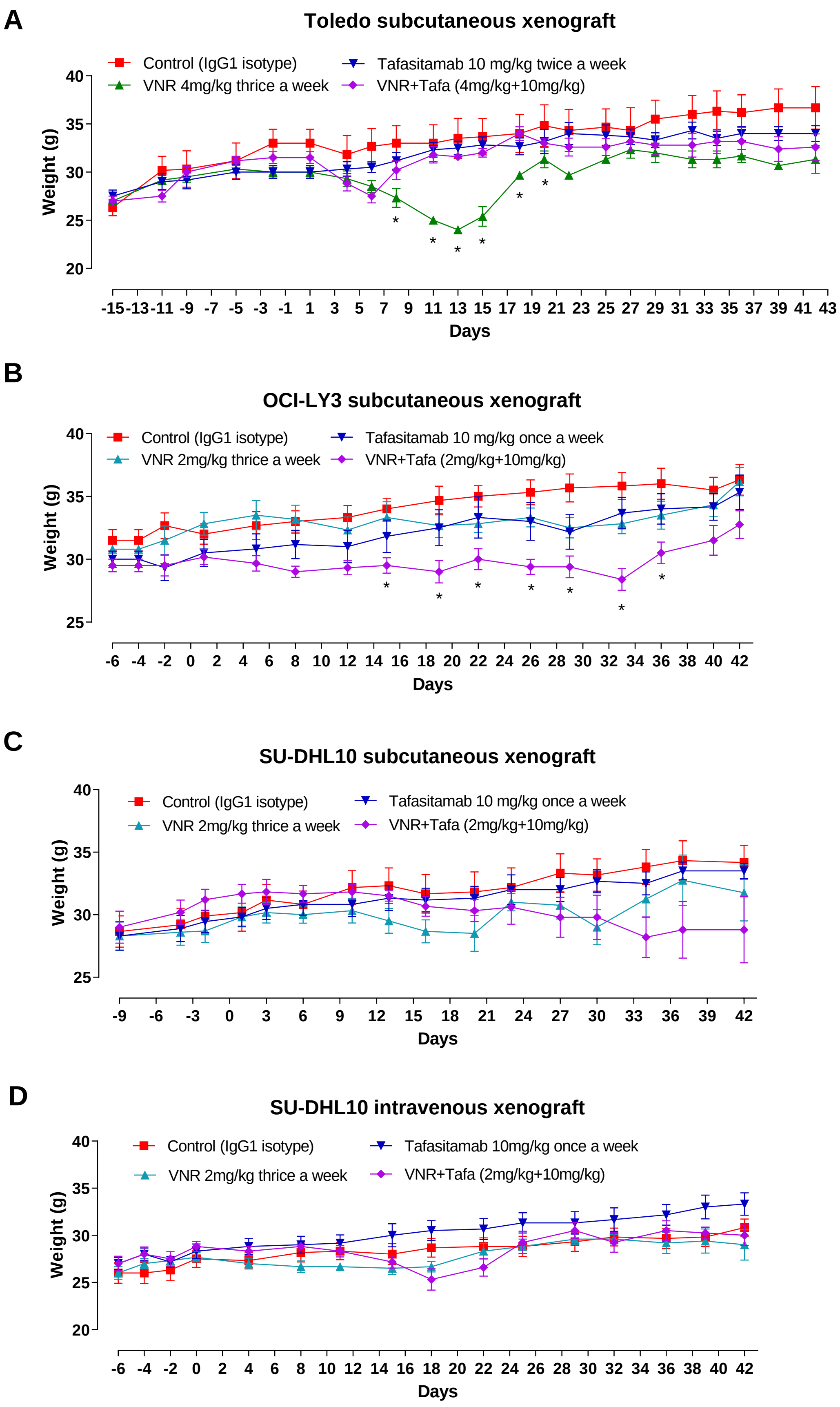

Figure S8. Weights of CD nu/nu mice bearing subcutaneous (**A**) Toledo, (**B**) OCI-LY3 and (**C**) SU-DHL10 xenotransplants and systemic (**D**) SU-DHL10 xenotransplant, recorded during the 42 days of treatment.

Table S1. Dose reduction index values for the drugs combination at 30%, 50%, 70% and 90% levels of inhibition of Toledo, OCI-LY3 and SU-DHL10 cell viability after 144 h of metronomic vinorelbine (VNR) plus metronomic etoposide (ETO) plus tafasitamab (TAFA) concomitant combination treatment.

Fraction affected	Dose Reduction Index								
	Toledo			*OCI-LY3*			*SU-DHL10*		
	VNR	TAFA	ETO	VNR	TAFA	ETO	VNR	TAFA	ETO
**30%**	0.81	1.53	0.92	26.37	315.63	9.53	150.41	4.38	110.92
**50%**	0.85	4.43	1.02	32.03	314.53	43.61	135.49	17.86	115.9
**70%**	0.9	12.87	1.11	38.91	313.44	199.56	122.06	72.75	121.1
**90%**	0.98	70.73	1.30	53.04	311.7	2250.82	103.35	681.79	129.88
